# Unveiling the Relation between Cellular Aging, Epigenetics and Cancer

**DOI:** 10.14336/AD.2025.0677

**Published:** 2026-06-26

**Authors:** Pawel Kordowitzki, Arkadiusz Grzeczka

**Affiliations:** ^1^Department of Basic and Preclinical Sciences, Nicolaus Copernicus University, Toruń, Poland; ^2^Department of Gynecology including center of oncological surgery, Charite, Berlin, Germany

**Keywords:** cellular aging, senescence, epigenetics, DNA methylation, cancer, tumorigenesis

## Abstract

This Editorial article intends to unravel the relationships among cellular aging, epigenetic changes, and tumorigenesis, thereby offering perspectives that could improve therapeutic approaches in cancer management and promote future research on these topics. Furthermore, selected fundamental principles concerning cellular aging will be presented to elucidate how this process contributes to the comprehension of tumorigenesis. As humans age, there is a progressive decline in physiological functions, which significantly increases the risk of cancer. Epigenetic alterations—heritable yet reversible modifications of the genome without changes in DNA sequence—play a pivotal role in both aging and tumorigenesis. Age-associated epigenetic drift, involving widespread DNA methylation changes, histone modification shifts, and chromatin remodelling, disrupts normal gene regulatory networks, leading to genomic instability and impaired cellular homeostasis. Additionally, the accumulation of senescent cells, driven by epigenetic dysregulation, fosters a pro-inflammatory environment that can promote tumorigenesis. Moreover, the epigenetic landscape of aged tissues resembles that of cancerous tissues, suggesting that aging establishes a permissive environment for malignant transformation. Understanding the interplay between aging, epigenetic regulation, and cancer is critical for the development of preventive strategies and novel therapeutics. Epigenetic reprogramming technologies, aiming to restore youthful epigenetic states, hold promise for delaying aging and reducing cancer incidence. However, challenges remain in selectively targeting pathogenic epigenetic changes without disrupting essential cellular functions.

The world is facing a multi-faceted crisis that encompasses challenges of an aging population. The intersection of cellular aging, epigenetics, and cancer has become a focal point for researchers aiming to unravel the complexities of oncogenesis. For 2025, it is anticipated that there will be 2,041,910 novel cancer diagnoses in the United States. Cancer ranks as the second predominant reason for mortality in the United States overall and as the primary cause among individuals younger than 85 years [1]. Cancer can potentially emerge across the duration of life in humans; however, approximately fifty percent of all neoplasms are recognized in patients over 70 years of age. Due to the overall tumor prevalence escalating with advancement in age and aging being the most significant risk element for cancer, it is typically regarded as an age-related condition [2]. In consequence, one could hypothesize, that if cancer is the shadow of aging, epigenetics may be the lens that ultimately brings it into focus. Interestingly, we are on the verge of a revolutionary period in geroscience and oncology, during which the methylome may compete with the genome in terms of therapeutic and predictive capability. In this Editorial we intend to emphasise that age is more than just a number; it's a molecular story told through changing methylation patterns, muted tumour suppressors, and the inflammatory trace of senescent cells.

In consequence, understanding the specific epigenetic changes occurring in aged cells is vital, particularly those linked to an elevated cancer risk, as these alterations can significantly influence oncogenic processes [2]. Cellular aging refers to the gradual decline in cellular function and regenerative capacity over time. This phenomenon is intricately linked to various epigenetic modifications, which are the chemical changes to DNA and histone proteins that regulate gene expression without altering the DNA sequence itself [3, 4]. Other epigenetic changes, such as chromatin remodelling and non-coding RNAs, are considered hallmarks of aging [5]. Some histone modifications act as switches for aging processes, suppressing genes that induce aging. Polycomb histone methyltransferase enhancer of zeste 2 (Ezh2) controls the level of histone H3 methylation at lysine 27 (H3K27me3), and deletion of the Ezh2 gene causes premature aging of mesenchymal cells [6,7]. Interestingly, in human nucleus pulposus tissues, Ezh2 controlled the cGAS/STING pathway, and its reduced expression in aging nucleus pulposus cells promotes inflammation associated with aging and the progression of intervertebral disc degeneration [8]. In turn, the acetylation of certain histones (HDAC4, HDAC5, HDAC6) at gene promoter sites is associated with reduced telomere length [9], and H3K14 acetylation is essential for the maintenance of heterochromatin and DNA repression [10]. For example, acetylation or mutations in the N-terminal tail of histone H3 at position K14 prolong replication lifespan and aging [10]. Epigenetics play a key role in mammalian development, influencing gene regulation and genome stability, but undergoes significant demethylation at key moments such as germ cell formation and fertilisation [11]. Changes in DNA methylation patterns are also associated with the aging process - with age, there is both a global loss of methylation and local hypermethylation, which can affect cell function and increase the risk of age-related diseases. Osteoporotic changes, characteristic of elderly people and those with reduced physical activity, are a consequence of bone homeostasis disorders. This is controlled, among others, by the H19 gene. Hypermethylation has been detected at the H19 promoter site in association with reduced bone density [12]. DNA hypermethylation was also found in some cells of the aorta affected by atherosclerotic changes [13]. In addition, there are some indications that environmental pollutants, such as heavy metals and persistent organic compounds, may disrupt this aging process and accelerate age-related epigenetic changes [14]. Therefore, further research is needed to clarify how external factors influence epigenetics, whether they can modulate the rate of aging, and whether these changes can lead to specific health outcomes, including the development of cancer. This also raises a new important question: Do scientists and researchers have the courage to read and rewrite old paradigms?

We hypothesise that cancer could be redefined as an epigenetic result of uncontrolled biological aging rather than just as a genetic disorder. This raises another radical question: would it be possible to postpone or avoid cancer by modifying epigenetic changes? Senolytic approaches and targeted epigenetic reprogramming could be core tenets of a revolutionary, age-informed oncology rather than marginal ideas. If we understand why centenarians are able to avoid cancer despite their biological deterioration, could we instill resilience in the rest of humanity? This is not only a plea for research; it is also a plea to reimagine intervention and the aging process. With the help of this Editorial, we aim to stimulate new research that will lead to a paradigm shift, meaning preventing disease at its epigenetic origin rather than treating it.

Research suggests that specific epigenetic modifications play a critical role in age-associated changes in cells [15, 16]. These modifications can lead to the dysregulation of tumor suppressor genes, and in consequence, the activation of oncogenes, creating a favorable environment for cancer development [17-19]. The convergence of epigenetic changes in aging and cancer is a series of changes leading to the destabilisation of genetic material, the inhibition of tumour suppressor genes and the activation of oncogenes, and the creation of a microenvironment conducive to cancer. For instance, in normal gastric mucosa, an age-related increase in methylation of suppressor gene promoters (LOX, p16/CDKN2A, RUNX3, TIG1) is observed, which may indicate the existence of subpopulations of cells with altered epigenetics, predisposed to malignant transformation [20,21]. Reduced DNA methylation leads to genomic instability through the activation of normally silenced sequences, such as retrotransposons, and disrupts chromatin structure, increasing susceptibility to mutations and chromosomal rearrangements characteristic of the carcinogenesis process [22]. Moreover, a global DNA hypomethylation in leukocytes is significantly associated with an increased risk of bladder cancer, as confirmed in a study comparing 775 cases with 397 controls. Patients with the lowest methylation levels (3.03%) had a 2.67-fold higher risk of developing the disease than those with the highest levels (3.19%). Importantly, this association was independent of smoking, as even in non-smokers, low methylation levels were associated with a 6.39-fold increase in risk, suggesting that global DNA hypomethylation may serve as a biomarker predisposing to cancer development [23]. At the same time, some elements are hypermethylated, which is associated with reduced transcriptional availability of genes responsible for protection against cancer [24]. For example, in colorectal cancer, CDKN2A and MLH1 are hypermethylated [25]. Aberrant methylation of microRNAs, such as mir-9-1, is an important mechanism of its inactivation in breast cancer, observed in as many as 34-86% of cases [26]. This phenomenon, which is an early and common stage of tumour development, correlates with the methylation of classic tumour suppressor genes (RASSF1A, cyclin D2, DAP kinase, SOCS-1) and may lead to the inhibition of the expression of genes with anti-tumour functions [27]. In colorectal cancer, miR-124a is a molecule subject to hypermethylation. Loss of miR-124a expression results in the activation of oncogenic CDK6 and phosphorylation of the RB1 protein, which promotes neoplasia [28]. In terms of histone modifications, the effects of global loss of H4K16ac and H4K20me3 accumulate [29], with a decrease in the activity of acetyl transferases and deacetylases that control the level of histone acetylation, contributing to DNA repair disorders, decreased DNA stability and loss of heterochromatin [30, 31].

Contemporary research provided evidence that aging is a multifaceted biological process characterised by progressive changes at molecular, cellular, and tissue levels, leading to a decline in physiological function [32]. Within oncology, this age-related trajectory poses significant implications for tumor biology, particularly in how it reshapes the tumor microenvironment. Interestingly, one of the key biological mechanisms by which aging alters the tumor microenvironment is through the processes of cellular senescence and the resulting inflammatory responses [32]. As cells age, they enter a state of senescence, characterized by permanent cell cycle arrest and changes in their secretory profiles. These senescent cells often exhibit a senescence-associated secretory phenotype (SASP), which compromises surrounding tissue and influences immune responses [33]. In this regard, we hypothesize that the timing of epigenetic changes, like miRNA silencing could be used as a predictive clock for cancer transformation.

Furthermore, the SASP releases pro-inflammatory cytokines, growth factors, and proteases, which not only create a pro-tumour environment but also facilitate the recruitment of immune cells that can support tumour growth rather than suppress it. Hence, this interplay can create a microenvironment that is more conducive to tumor progression rather than suppression [33, 34]. Recent studies have shown that senescent cells can alter the extracellular matrix and attract immune cells, thus transforming the microenvironment in ways that can enhance tumor growth and invasion [35, 36]. Although individual cancers, such as ovarian, liver, and stomach cancer, are characterised by different growth dynamics and depend on different molecular pathways, the primary tumour microenvironment, associated with the activity of aging cells, shows striking similarities in many types of cancer. For instance, in aging ovaries, collagen and fibronectin accumulate, which, together with elevated levels of IL-6, TNF-α and IL-1β, promote chronic inflammation [37, 38]. Activation of TGF-β and NF-κB pathways induces EMT and EndoMT transitions and activation of fibroblasts to the CAF (cancer-associated fibroblasts) phenotype. Translationally, an interesting avenue in our opinion could entail the partial reprogramming of old somatic cells to recover youthful epigenetic signatures without erasing cellular identity, using CRISPR-based epigenome editors and other similar tools. This could postpone oncogenic epigenetic remodelling, especially in high-risk areas like the liver or ovary.

Moreover, macrophage infiltration, especially of the M2 type, enhances immunosuppression and secretes factors (e.g. TGF-β, IL-10) that enhance EMT and CAF activation [39]. Overall, these processes promote angiogenesis, matrix degradation by MMPs and increased invasiveness of ovarian tumours, increasing their malignancy. With age, the liver undergoes a series of changes that promote the progression of fibrosis [40]. Senescent cells (p21^+^, γH2AX^+^) accumulate, strongly secreting cytokines, chemokines (CCL2) and MMPs, creating inflammation and activating stellate cells [41]. Activated HSCs differentiate into collagen-producing myofibroblasts, thereby promoting carcinogenesis [42]. In the aging liver, the activity of repair pathways such as AMPK and SIRT1 is reduced, and ER stress, mitochondrial dysfunction and oxidative stress are increased [43, 44]. In turn, impaired proteostasis and autophagy result in impaired toxin removal, which exacerbates fibrosis and impairs hepatocyte regeneration. All these changes together create an environment conducive to both fibrosis progression and the development of hepatocellular carcinoma (HCC) [42].

As populations age, understanding the nexus between aging and cancer becomes increasingly vital, given that cancer is predominantly a disease of older individuals [45, 46]. As people age, they become more susceptible to chronic, non-communicable diseases such as cancer. According to the World Health Organization, the rapid growth of the older population, projected to increase from 10% in 2022 to 16% by 2050, is expected to further exacerbate this challenge, requiring healthcare systems to adapt and evolve in order to effectively manage the complex needs of the aging population [47]. Supercentenarians are often viewed as the epitome of successful aging, possibly due to unique qualities contributing to their extended lifespans. While the media frequently attributes specific reasons to their longevity, limited research exists on the qualitative aspects reported. Therefore, it would be interesting in future research to unveil the molecular pathways and properties of centenarians which appear to protect them from cancer. To date, few genes have been identified that are indisputably associated with longevity. Another interesting route that is worth exploring in our eyes is the description of epigenetic resilience in centenarians, who show resistance to age-related hypermethylation of tumour suppressors. This could potentially shed light on protective regulatory variants or metabolite profiles.

Former research provided evidence that the association between the APOE4 allele and longevity is clearly negative, as confirmed by population studies. Carriers of the APOE4 allele (especially homozygotes) have a higher risk of overall mortality (HR 1.63 for homozygotes) and cardiovascular disease [48]. In addition, individuals with the APOE-ε4 allele have significantly shorter telomeres than individuals without this variant. What is more, higher concentrations of glucose, glycohexose and fluorescent AGEs in plasma correlate with greater telomere shortening and may link genetic risk (APOE-ε4) to metabolic processes, increasing susceptibility to Alzheimer's disease [49].

**Figure 1.**
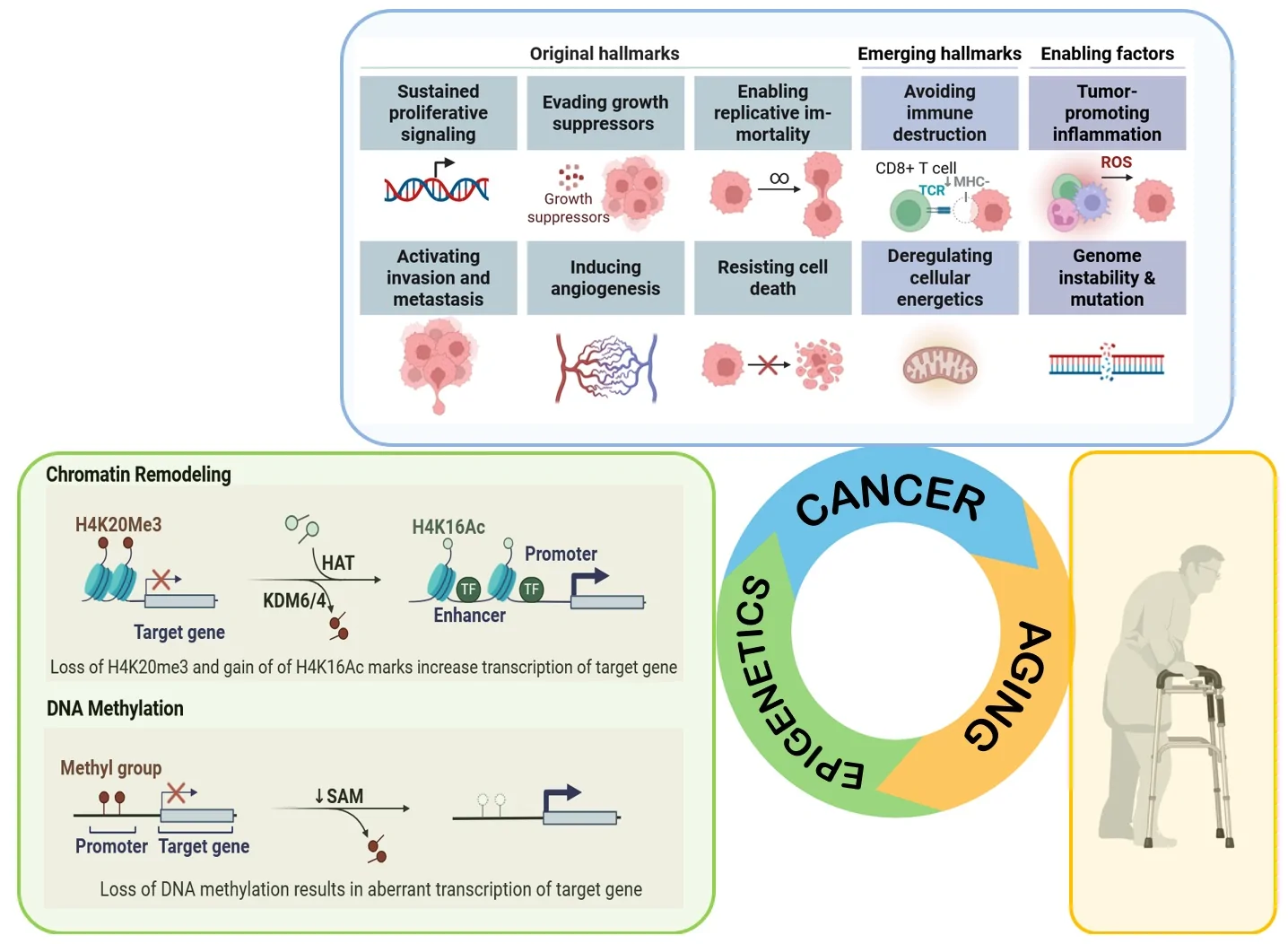
Scheme showing the relation between aging, cancer, and epigenetics.

Another promising candidate is FOXO3, whose variants are associated with longer life and improved resistance to cellular stress and inflammatory processes. Key pathways associated with longevity include insulin/IGF-1 signalling, DNA repair, immune function (IL6), cholesterol metabolism (APOE) and telomere maintenance (POT1) [50]. The accumulation of cellular damage, alterations in cell signalling, cell death, and the resulting modifications in the extracellular environment can profoundly influence tumour behaviour and treatment responses [51, 52]. Importantly, cell death plays an important role in tumour development and biology, since by this, senescent or damaged cells are eliminated [53]. Typical cancer treatments can lead to cell death and help reduce tumours, but cancer cells may become resistant to these treatments and withstand them [54]. Resistance to apoptosis is both a characteristic of aging cells and a key mechanism underlying cancer resistance to treatment. Cancer cells often acquire mutations or epigenetic modifications that disrupt apoptotic pathways, such as overexpression of anti-apoptotic proteins from the BCL-2 family or loss of p53 protein function [55]. This leads to the ineffectiveness of conventional therapies, including chemotherapy and radiotherapy, which act mainly by inducing apoptosis [56]. New research indicates that cancer cells can activate alternative survival pathways, such as NF-κB, mTOR or PI3K/AKT, which further complicates treatment [57-59]. Overcoming this resistance, e.g. through BCL-2 inhibitors or modulation of immunogenic cell death, is currently an important area of research [60].

Furthermore, identifying the epigenetic modifications related to tumorigenesis has become a key area of study in cancer research, with the aim of elucidating how the epigenetic control of cancer cells works. In reality, various phases of tumour development, such as the formation of tumours, their growth, and advancement, are often linked with epigenetic changes [61]. In consequence, these epigenetic modifications can alter cellular functions and, in consequence, contribute to the aging process [62]. The interplay between aging and epigenetics may disrupt normal cellular mechanisms, promoting tumorigenesis (Fig 1).

By investigating how epigenetic alterations accumulate with age, researchers may identify epigenetic biomarkers for early cancer detection. Moreover, targeting these epigenetic modifications might enhance the effectiveness of existing therapies and lead to more personalised approaches in oncological care. Continued research is essential to decipher the complex mechanisms by which aging and epigenetic factors converge in cancer progression and could also provide strategies for healthy aging and cancer prevention. In aged cells, hypermethylation of tumor suppressor genes are common, destabilizing genomic integrity and enhancing susceptibility to malignancies. Analyses have shown that specific methylation patterns, particularly in CpG regions of genes associated with cancer (such as C1q13, Srd5a2 and Ptk7), are highly conserved evolutionarily. Furthermore, a clear correlation has been observed between the aging process and the intensity of methylation in these areas of the genome [63]. For example, in adenocarcinomas, the hypermethylation of genes such as p16INK4A, p14ARF and APC serves a dual role by promoting cellular senescence while simultaneously influencing oncogenic pathways, emphasizing the complex interplay between aging and cancer [64].

Understanding these mechanisms may also facilitate the design of drugs that reverse harmful epigenetic changes. It is worth mentioning that the potential to reverse age-related epigenetic alterations through targeted therapeutic strategies is an area of growing interest. These epigenetic interventions have been shown to restore normal cell function, thereby reducing the cancer risk associated with aging. For example, inhibitors of DNA methyltransferases and histone deacetylases have demonstrated promise in preclinical models by reversing aberrant gene expression patterns linked to senescence, offering a pathway towards mitigating age-associated cancer risk [65,66]. However, the aforementioned small-molecule drugs that target epigenetic alterations also have several obstacles. These include the potential for relapse caused by the reversibility of epigenetic changes, the challenges of delivering medicines to particular tissues, and the lack of specificity, which can result in toxicity and off-target effects [67,68].

Therefore, this editorial advocates for integrating insights from aging biology and cancer genetics with the aim that researchers can pioneer innovative treatments that address the root causes of tumor formation, improve risk assessments, and introduce tailored interventions for at-risk populations. Ultimately, bridging the gap between epigenetics and aging may not only enhance our understanding of cancer biology but also foster advancements in preventive health strategies that promote longevity and reduce the burden of cancer. This interdisciplinary approach could revolutionize public health initiatives and change the landscape of cancer care. By emphasizing prevention and early intervention, we can shift the focus from treatment to maintaining health. This paradigm shift may lead to more effective strategies that address the underlying causes of disease, enhancing quality of life for aging populations. It may also encourage lifestyle modifications and environmental changes that mitigate cancer risk. However, despite the burgeoning interest in the relationship between aging and epigenetics in cancer development, significant methodological limitations persist in current research. Numerous studies rely solely on cell lines or animal models that may not accurately reflect human aging processes. Additionally, the heterogeneity of cancer types and the varying impact of aging on epigenetic regulation complicate the ability to draw definitive conclusions across different types of cancer. Emerging studies show disparate results concerning age-related epigenetic regulation, highlighting the need for larger-scale, multi-center studies that encompass a diverse array of cancer types to refine these findings. Finally, environmental epigenetics presents an underexplored frontier: investigating whether lifestyle changes, such as detoxifying pollutants or mimicking caloric restriction, can reverse prooncogenic methylation changes offers a preventive strategy with wide public health implications. These ideas need to be thoroughly validated in longitudinal, multiomics studies involving varied aging populations.

## Conclusions and outlook

Future research endeavors must also tackle the causation versus correlation dilemma inherent in studies examining the interplay between aging, epigenetics, and cancer outcomes. A robust framework integrating multidisciplinary approaches could better elucidate the biological underpinnings of these relationships. Employing advanced technologies such as single-cell sequencing and epigenome editing is pivotal for parsing out the intricate causal mechanisms linking aging to cancer. In our opinion, the following important points should be addressed in future research:

1. Embrace multidisciplinary frameworks that integrate molecular biology, oncology, and geroscience to analyze causal mechanisms.
2. Utilize cutting-edge technologies, particularly single cell sequencing and epigenome editing, to elucidate cell-specific epigenetic changes occurring during aging and tumorigenesis.
3. To improve translational relevance and validate findings, conduct large-scale, multicenter studies across various populations and cancer types.

These steps are crucial for creating accurate, age-informed cancer treatments that consider senescence's dual roles in promoting and inhibiting tumors. As we consider the implications of immune system alterations with age, addressing how these changes impact cancer treatments is vital. In summary, the nuanced role of cellular senescence in cancer progression underscores its complexity as both a barrier to and a facilitator of tumorigenesis.
